# REPRODUCIBILITY OF SCHATZKER CLASSIFICATION THROUGH SMARTPHONE APPLICATIONS

**DOI:** 10.1590/1413-785220162406159078

**Published:** 2016

**Authors:** Mauro Rodrigues dos Santos, Junichiro Sado, Roberto Medeiros de Sousa, Osman Rodrigues Roriz

**Affiliations:** 1. Universidade Federal de Goiás, Hospital das Clínicas, Department of Orthopedics and Traumatology, Knee Group, Goiânia, GO, Brazil.

**Keywords:** Tibial fractures/classification, Tibial fractures/radiography, Cell p. hones/utilization, Mobile applications/utilization

## Abstract

**Objective:**

To evaluate the intra-observer reproducibility of Schatzker classification for tibial plateau fractures through smartphone applications.

**Methods:**

Radiographs were evaluated in two incidences (anteroposterior and profile) and CT slices (axial, sagittal and coronal) of 37 patients with tibial plateau fracture. Two evaluators, knee surgery experts, classified the cases by viewing the images of the isolated radiographs and then X-rays associated with CT slices in four different stages via smartphones and then presential assessment. Data were statistically analyzed with the Kappa coefficient (k).

**Results:**

There was intra-observer agreement by comparing the two methods of evaluation: display or via smartphone, and the analysis made showed statistical significance.

**Conclusion:**

The use of smartphones did not affect the reliability of Schatzker classification. Level of Evidence III, Diagnostic Study - Investigating a Diagnostic Test.

## INTRODUCTION

Tibial plateau fractures are common injuries that include a wide range of morphological patterns. A number of classification systems have been proposed to categorize these types of fractures, in order to simplify communication in clinical practice, to provide guidelines for preoperative planning, and to allow comparison with the data presented in the literature. The three classification systems used in the assessment of tibial plateau fractures include the OTA/AO system, the Schatzker classification and Hohl's classification.^1^ ^-^ ^3^

X-rays images and CT scans are commonly and frequently shared through smartphone devices between orthopedic surgeons and fellow residents, a technological facility that allows the debate at any time with team members who are far away from each other. Obviously, it does not replace the presential assessment and care provided to the patient by a qualified professional.

Currently, there are numerous software applications that can be installed on smartphones. Health-related applications are increasingly being developed, with approximately 1,000 new applications launched every month and 142 million downloads annually, estimated by 2016. Its use is popular, with 85% of medical professionals using smartphones and 30-50% using applications in clinical practice. The range of applications available for smartphones has been reported in various specialties, including orthopedics, colorectal surgery, anesthesiology, radiology and microbiology.^4^ ^-^ ^9^ However, concerns regarding the lack of physician's involvement in developing the application and the reliability of the application's content have been raised.

The use of smartphones applications in orthopedics have been described in the literature, such as measuring the angular deviation in hallux valgus^4^ ^,^ ^5^ and spinal deformities,^6^ measuring the flexion-extension after arthroplasty^9^ and also the use of these applications in radiology.^8^ ^,^ ^10^ ^-^ ^12^

The aim of this study is to evaluate the intra-observer reliability of the Schatzker classification for tibial plateau fractures using smartphones. 

## MATERIALS AND METHODS

We retrospectively evaluated 37 cases of tibial plateau fractures treated at the same hospital between 2011 and 2014. All patients had knee radiographs in anteroposterior (AP) and lateral (L) views supplemented with computed tomography (CT). All these tests were stored in digital media.

The two evaluators were orthopedic surgeons, members of the Brazilian Society of Orthopedics and Traumatology (SBOT) and the Brazilian Society of Knee Surgery (SBCJ) with expertise in the treatment of tibial plateau fractures. They will be hereafter designated as evaluator 1 and evaluator 2.

In the first stage, all AP and L radiographs were photographed using a smartphone and sent, case by case, in random and independent order to smartphones of the two evaluators using the mobile application Whatsapp^(r)^. Then, they applied the Schatzker classification and sent the evaluations to the examiner, which, in turn, sent the photos of other cases to the evaluators, successively, until completion of the 37 cases.

The second stage was carried out similarly to the first, in random and independent order, however, in addition to the X-rays, CT images in coronal, axial and sagittal views were added to each evaluated case.

In the third and fourth steps, performed six months after the first, the same cases and images were rated by two evaluators in presential way, directly from the hospital's computer monitor. In the third stage, the examiner presented only the X-ray image, and in the fourth stage, X-rays and CT scans.

The smartphones used for both capturing and sending images and classifying images by the evaluators were Apple^(r)^ iPhone 5S model with 4-inch screen. Regarding the presential assessment, the images were displayed on a full HD 21.5-inch monitor (1920 x 1080 pixels), and the software DViewer was used to visualize the images. Both the computer as the smartphone software allowed magnification of the image on their screens.

Data were entered and manipulated in Excel charts, for further processing using the Statistical Package for Social Sciences (SPSS) for Windows (version 21.0).

For concordance analysis was used the Kappa test. The values ​​of this index ranged from +1 (perfect agreement) through zero (fortuitous or chance agreement) up to -1 (no agreement). In general, values ​​lower than 0.5 were considered unsatisfactory, values between 0.5 and 0.75 were satisfactory, and values higher than 0.75 are considered excelent.^13^

For all tests we used a 95% level of confidence, i.e., p<0.05 was considered statistically significant. This study, that dealt with analysis of medical records, did not require approval by the Ethics Committee.

## RESULTS

Both evaluators showed significant agreement, with Kappa above 0.7 and p <0.001, in X-ray only presential and smartphone intra-observer evaluations. (Table 1)

**Table 1:** Concordance analysis of the results of X-ray observations in presential way and using smartphones.

Observer	Concordance		Discordance		Kappa	*p*
	N	%	n	%		
Evaluator 1	30	81.1	7	18.9	0.77	< 0.001
Evaluator 2	29	78.4	8	21.6	0.73	< 0.001

Regarding presential and smartphone evaluation of X-ray image and CT scan, we observed that despite the evaluator 1 showed Kappa 0.68 and the evaluator 2 Kappa 0.81, both had very good and significant agreement with Kappa above 0.6 and *p*<0.001. (Table 2)

**Table 2:** Concordance analysis of the results of X-ray plus CT-scan observations in presential way and using smartphones.

Observer	Concordance		Discordance		Kappa	*p*
	N	%	n	%		
Evaluator 1	28	75.7	9	24.3	0.68	< 0.001
Evaluator 2	32	86.5	5	13.5	0.81	< 0.001

The analysis of observations together by both evaluators, both presential as smartphone evaluations of x-ray alone or together with CT scan showed significant agreement, with Kappa above 0.7 and p<0.001. (Table 3)

**Table 3:** Concordance analysis of the results of observations by evaluator 1 + evaluator 2 in presential way and using smartphones.

Method	Concordance		Discordance		Kappa	*p*
	N	%	n	%		
RX	59	79.7	15	20.3	0.75	< 0.001
RX + TC	60	81.1	14	18.9	0.75	< 0.001

## DISCUSSION

A common and frequent practice among orthopedic surgeons and residents is to share radiological images and tomography scans by electronic means. However, this practice requires scientific validation, since it is not known whether this technology may lead to mistaken interpretation of the images, and, consequently, improper decision making by the surgical team.

In this study, the application of Schatzker classification for tibial plateau fractures through images interpreted on smartphones screens *versus* presential screening in intra-observer analysis was considered satisfactory or excellent, with all indices above 0.5, the largest 0.81 and the lowest 0.68.

It should be noticed that the two evaluators are orthopedic surgeons experienced in the treatment of tibial plateau fractures, which enhanced the degree of agreement. In a study by Albuquerque et al.,^14^ it has been shown that inexperienced surgeons tend to have lower intra- and inter-observer variability for the different classifications of tibial plateau fractures (Hohl, AO or Schatzker). Therefore, we chose to employ experienced surgeons in this work, minimizing the effects of this important variable, enabling us to focus specifically on possible influences of smartphone devices on the results.

Other studies have observed unsatisfactory K indices (near 0.38) in inter-observer analysis^2^ ^,^ ^15^ and satisfactory kappa values (0.68) in intra-observer analysis^2^ for this classification, which makes clear that the correlation value decreases when the evaluation is between the evaluators. In this study, the concordance results are similar to results obtained in intra-observer analysis for Schatzker classification.

## CONCLUSION

The use of images through smartphone devices did not interfere with the intra-observer agreement of Schatzker classification of tibial plateau fractures in this study. The same result was obtained to rank X-rays only and also X-rays combined with tomography scans.
